# mHealth and e-Learning in health sciences curricula: a South African study of health sciences staff perspectives on utilisation, constraints and future possibilities

**DOI:** 10.1186/s12909-023-04132-4

**Published:** 2023-03-28

**Authors:** Habib Noorbhai, Tinuade Adekunbi Ojo

**Affiliations:** Biomedical Engineering and Healthcare Technology (BEAHT) Research Centre, Faculty of Health Sciences, University of Johannesburg, Johannesburg, South Africa

**Keywords:** mHealth, e-Learning, Health sciences curricula, Staff perspectives, 4IR

## Abstract

**Background:**

Over the last decade, developments in e-Learning and technologies are creating the groundwork for health sciences and medical education. Literature demonstrates that we have yet to reach any form of consensus about which indicators are needed to assess and teach quality health sciences and medical education through technology or innovation. There is, therefore, a greater need for a tool or platform that is properly constructed, validated and tested within health sciences.

**Methods:**

This paper presents a study, which is part of a larger research project assessing staff and students’ perceptions of the importance and relevance of different aspects of e-Learning and mHealth in health sciences curricula at four universities in South Africa. The specific objectives of this study were to: (i) assess health sciences staffs’ perceptions and understanding of these two applications; and (ii) establish challenges and opportunities of e-Learning and mHealth applications in the health sector, as well as perceptions on the importance and relevance of these applications to their curricula and future practices. A combination of Focus Group Discussions (FGDs) and a key-informant interview was used. A total of 19 staff from four universities participated. Atlast.ti was used for the data analysis and the findings were coded using a primarily deductive thematic coding framework.

**Results:**

The findings revealed that not all staff members are equipped or trained with new applications or technologies, such as mHealth. Most participants believed that diverse technologies and tools could be integrated with mHealth and e-Learning. Furthermore, participants agree that a new multi-modal platform, in the form of a learning management system (LMS) with relevant applications (and possible plugins) integrated, tailored towards health sciences will benefit all stakeholders, and be valuable to higher education and health sectors.

**Conclusions:**

Digitalisation as well as digital citizenship is gradually being integrated into teaching and learning. It is imperative to adapt the health sciences curricula through constructive alignments and promote health sciences education in the current 4IR. This would allow graduates to be better prepared for digitalised practice environments.

## Background

The health sector has witnessed several challenges in identifying and treating diseases in Africa. Scholars have debated the constraints affecting the health sciences, including the health faculties and medical schools in higher learning institutions of developing economies (low, middle-income countries [LMIC]) on addressing the burden of diseases, preventive medicine, and treatment due to inadequate facilities. Since the commencement of the Coronavirus-19 (COVID-19) pandemic, South Africa has witnessed the evolution of different conditions such as human immunodeficiency virus/acquired immunodeficiency syndrome and tuberculosis, diabetes, maternal and child mortality, injuries and violence, and non-communicable diseases amongst many [1, 2]. Understanding the relationships between ecosystem health and human health is paramount despite this epidemic and the epidemiology of diseases globally. Most significantly, it is recorded that the practice of medicine is more than identifying and treating disease; it includes a significant preventative component which requires innovative health care approaches, especially in limited-resource settings [3]. However, teaching and learning for this context have become a challenge due to the already overloaded curricula and inadequate infrastructures [4].

In addition, most South African health sciences faculties within universities are resource-constrained and do not foster a dynamic e-Learning and mobile health (mHealth) culture within their curricula. mHealth focuses on obtaining information immediately from mobile phones to diagnose illnesses, track diseases and provide timely information to the public in underserved countries. In addition, mHealth can also be used as a form of education for health sciences students. Embracing such a culture would create more job creation opportunities within the healthcare landscape. Some universities in South Africa, such as the University of Johannesburg, have started to integrate the ethos of the Fourth Industrial Revolution (4IR) but are yet to teach e-Learning (fully) within their health sciences curricula (and many other faculty curricula).

This paper aims to present health science staffs’ understanding and perceptions of the use, constraints and future possibilities of e-Learning, mHealth in their curriculum, which could significantly impact their future healthcare learning and practice post-COVID-19. In addition, given the unsuccessful rate of e-Learning or slow uptake within the health sciences area, the article presents the findings from in-depth interviews and focus group discussions from health sciences staff at four South African universities regarding the use and related constraints of mHealth and e-Learning applications, as well as possibilities for a multi-modal platform and curricula, in order to optimise learning (for students and academic staff) and healthcare (for patients) in South Africa.

### Theory and context of the literature

#### The concept of e-Learning

There appears to be no widely accepted definition of e-Learning. However, there is a convergence of describing what it entails: learning using e-Learning infrastructure, made up of both hardware and software, via various electronic devices and communication applications, requiring web/internet access. This form of learning can either fully replace the physical classroom (distance learning) or augment classroom-based teaching and learning and has come to be known as blended learning [5].

#### e-Learning in South Africa

The pandemic came with a significant shift in higher learning systems, demanding alternative and flexible teaching and learning approaches in the digitalised economy. This led to a call for easy access to online learning platforms that offer electronic assisted educational facilities [6]. In addition, most universities faced the challenges of integrating eLearning into the existing traditional offerings as a form of blended learning. As a result, most academics have found themselves within the digitalised space in which they are expected to adopt and implement different teaching and learning approaches. However, academic and administrative developments are expanding, including the changes that will facilitate South African universities’ strategic orientations and adoption of e-Learning.

The digital context in South Africa is characterised by initially slow uptake and limited device usage to rapid expansion from 2014 onwards. Market needs vary, and unique challenges regarding affordability about the costs of mobile technology and accessibility of the World Wide Web compared to data costs relating to other costs (electricity cost, transportation costs). The statistic shows that the number of internet users in South Africa from 2014 to 2018 increased from 9.7 million users to 20.3 million users [7]. However, these numbers grew to 38.13 million in 2021 and have increased in 2022. Initially, internet users ranged from the South African middle class to the top rank. However, all class sectors and societal structural levels have demanded flexible educational services [6]. The study relates with the ‘constructivist learning theory, which entails identifying learning processes within the classroom environment [8]. The idea signifies a cognitive activity that generates mental models that represent perceptions of reality. The theory focuses on answering questions on what people know and the reason for existence. The constructive approach assists academics in solving teaching, learning, and development [8]. This theory focuses on the belief that solving problems aids students and scholars in thinking, education, and development. The approach allows envisaging the different unique experiences and expertise of the staff interviewed and proposes solutions for future learning engagements [8].

#### e-Learning and classroom training

“Your schedule, pace, and place are the e-Learning motto” [9]. This is unfortunately not the case with classroom training. Contemporary studies have shown that the advantages of e-Learning compared to classroom training are emerging as wide-ranging. For example, e-Learning students retain the material significantly more than those in face-to-face instructor-led classes [9]; perform better than those who take contact courses [10] and demonstrate better comprehension [11]. Qazi et al .[12], further cite four benefits of e-Learning. The first refers to the issue of ‘cost difference’; e-Learning is cost-effective and affordable to many students, especially since universities are giving subsidies for data cost. Secondly, e-Learning is flexible and available 24/7, eliminating travel distance and enabling students to save costs. The third benefit entails reporting and monitoring; another benefit of e-Learning is the Learning Management System (LMS) which gives instant feedback and tracks the learner’s progress online without any additional administrative help. The fourth benefit involves consistency of e-Learning service delivery, where online learning students can access conveniently, as per demand, through varied learning resources [12].

Even so, quite a few higher education institutions have made limited use of e-Learning as part of their learning system. However, arguments have been made that the scarcity of teaching e-Learning at higher education institutions today could be insufficient capital, inadequate knowledge on access and usage, poor skills or any number of unknown factors withholding the adoption of e-Learning. Despite these challenges, there is a view that a combination of traditional classrooms and e-Learning creates entirely new experiences for learners, and new business opportunities for business owners by increasing the number of learners to educate and potentially addressing the low matriculation to degree qualification transition ratio [13]. The scholars agreed that the traditional form of learning and the e-Learning model benefits more shareholders within the educational setting and corporate environment [6].

The growth in e-Learning technology no doubt adds to globalisation as educational institutions are trying their utmost to break down geographical and social boundaries to offer distance learning education [14]. This leads to integrations of academic standards and views. Introduction of e-Learning to the business model provides diversity, flexibility and additional revenue streams representing a mixed or blended learning model. Students are expected to participate in different online activities in a hybrid learning or blended model. The online activities include discussions, online assessment, group work, and online projects replacing face-face teaching and learning [14].

e-Learning facilitates equitable and easy access to digital learning resources [14]. Despite being an additional learning resource that enhances access to learning tools, most families and teachers cannot afford the data necessary to sustain e-Learning activities [14].

#### mHealth to fill curricula gaps in the health sciences

Developments in e-Learning technologies have set the motion for a revolution in education within the last decade [15]. Evidence of these is seen within the health sector, where both intern and qualified doctors are frequently using mobile phones to consult with their patients. This can also be referred to as education, involving access to health education from a distance, using Information Communication Technologies (ICTs). Health practitioners benefit from the health education system through electronic libraries, search engines, and online knowledge databases [15]. Noorbhai [16] acknowledges the challenge health practitioners go through to streamline important information on symptoms, exposures, treatments, and various prevention strategies within the health sector. He postulated that streamlining this information is even more difficult within the current evolvement of research in the public health sphere. According to him, mHealth could be one of the solutions to address this issue.

Scholars have suggested some developments of mHealth, digital applications, and telemedicine, which have gained prominence and been used within the health sector, especially in developed economies [17, 18]. It was also showed that further studies are required to investigate the effectiveness of mobile applications on patients’ care and healthcare professional services [19]. However, there is already an overall positive impression of perioperative mHealth applications. The importance of further research was also substantiated regarding the role of telemedicine education on health professionals’ training, application and knowledge, including health stakeholders’ attitudes and practices [20].

### Research rationale

This research study is part of a broader research project that aims to establish optimised e-Learning platforms for health sciences students and academic staff, as well as exploring the integration of m-Health to better prepare students for their future work environments and enhance healthcare for patients in South Africa. The end goal of the larger project is the creation of a well-constructed platform that is validated and tested.

Given the limited success rate of e-Learning (uptake) within the health sciences field, this article aims to fill this gap by establishing staffs’ understanding of e-Learning and mHealth, their use within their curriculum, constraints and possible future developments.

Despite mHealth technologies’ impact on health research, it is assumed that the exponential growth of technology has outpaced the science of mHealth [21]. Since some health science curricula do not include mHealth training, health stakeholders do not have sufficient time to research the necessary new approaches and mediums; hence, further research needs to understand the capabilities and needs of students and staff stakeholders in the 4IR. In addition, an mHealth application that conforms with a robust network and promotes healthcare technology and innovation within the health sector should be developed, especially since there is an unsuccessful rate of e-Learning within the health sciences [22]. In addition, research on how platforms and curricula are established, merged and evaluated should be conducted to optimise learning and healthcare with patients and enhance quality assurance with students’ involvement. Furthermore, the report submitted by the World Health Organisation (WHO) supported the need for transformation and capacity training to strengthen the health workforce. However, the change will be enhanced if strategies are initiated to complement policies, retain graduates, as well as give students working conditions that promote knowledge and skills development. The proposed multi-modal platform (or tailored learning management system for health sciences) when being created would need to consider practical strategies that address the promotion of disease prevention as well as health sciences education.

## Methods

### Study design

The larger research project employs a modified Delphi method to realise five objectives in various phases. The main benefits of using a modified approach are that it enables contributions and building on previous work experiences in the field, irrespective of whether this has been published [23], as well as fostering co-operation [24]. This study reports on the first phase, entitled ‘Pre-survey FGDs (Expert Panel 1)’. The composition of the expert panel will be discussed below.

The qualitative method of FGD was deemed appropriate as a first phase to gather in-depth perspectives across institutional and disciplinary boundaries from experienced health sciences educators from which closed and open questions could be constructed for the subsequent phase. One interview was conducted with a key informant due to their unavailability to participate in FGDs. The shift to a quantitative method in the second phase, via a primarily inductively constructed survey questionnaire, would considerably increase the sample size of South African health sciences teaching staff. The benefit of employing qualitative methods prior to survey questionnaire design is that it contributes to reducing researcher bias inherent in closed questions, as well as building a community of practice in that staff in other universities and disciplines discuss how to make “tools of impact” that move beyond the university’s boundary within the health sciences.

The FGDs and interview addressed the following objectives within the larger project: (i) assess health sciences staffs’ perceptions and understanding of these two applications, as well their perceptions on the importance and relevance of these applications to their curricula and future practices, and ii) establish challenges and opportunities of e-Learning and mHealth applications in the health sector.

### Study participants

The expert panel is drawn from staff at four universities, some of whom are regarded among the top in South Africa, according to Times Higher Education rankings. They are the Universities of Cape Town (UCT), Witwatersrand (Wits), Johannesburg (UJ) and the Western Cape (UWC). The participants were from the following health professions/disciplines: medicine, physiotherapy, biokinetics, optometry and occupational therapy. The criterion for selection was that volunteer participants had to be a lecturer or teaching academic at the university within the health sciences.

Universities’ health science staff were contacted through internal networks and the Faculty Administration Offices. Permissions were obtained from the relevant personnel/research heads. The researchers ensured that all staff members understood the importance of voluntary participation and signed the consent form. All staff volunteers were included in the FGDs, and one was accommodated in an in-depth virtual individual interview. The line of questioning for the FGD and interview were the same.

### Study procedure

The total number of participants were *n* = 19. Virtual FGDs consisting of four expert panels (3 - 5 participants per panel) were conducted via Microsoft Teams or Zoom. Duration of each was 45minutes to 1 hour. These were augmented by one interview with a key informant from UCT.

All FGDs and the interview were conducted in 2021, within months of each other.

### Data analysis

Atlast.ti was used to analyse the responses from both the FGDs and the interview. Deductive thematic coding was applied as researchers used pre-formulated guide questions drawn from the literature to facilitate discussion in the context of the range and diversity of experience with mHealth and eLearning in South African Health Sciences Education. Participant responses were coded and synthesised into categories derived from the pre-formulated guide questions.

## Results

**Fig. 1:**
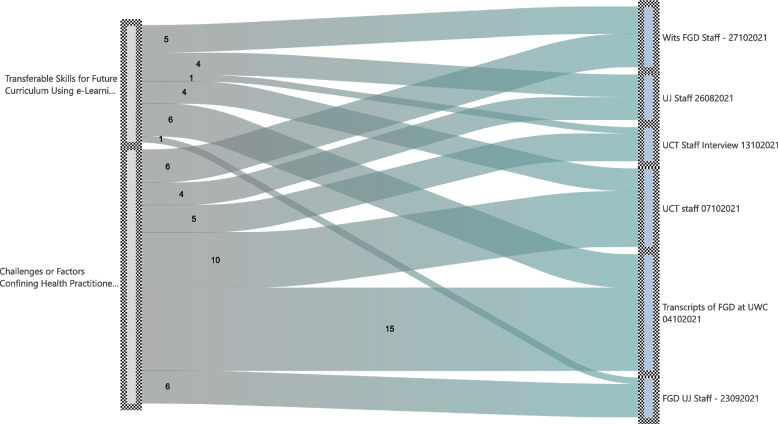
Perceptions and understanding of mHealth and e-Learning within Health Sciences Education

**Fig. 2:**
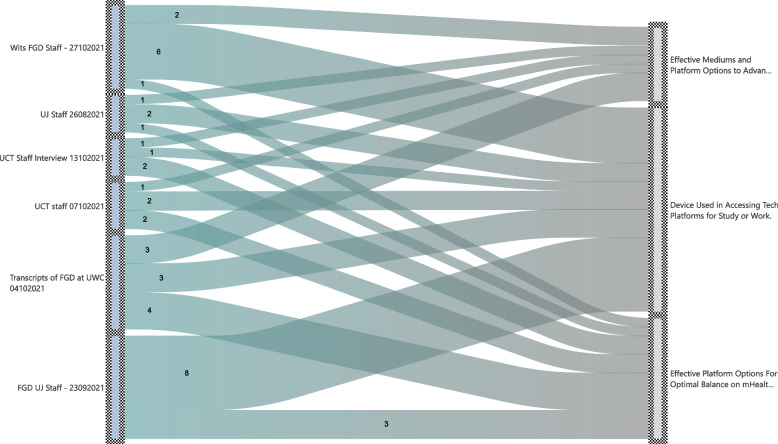
Effective Platforms, Devices and Mechanisms in Accessing mHealth and e-Learning

**Fig. 3:**
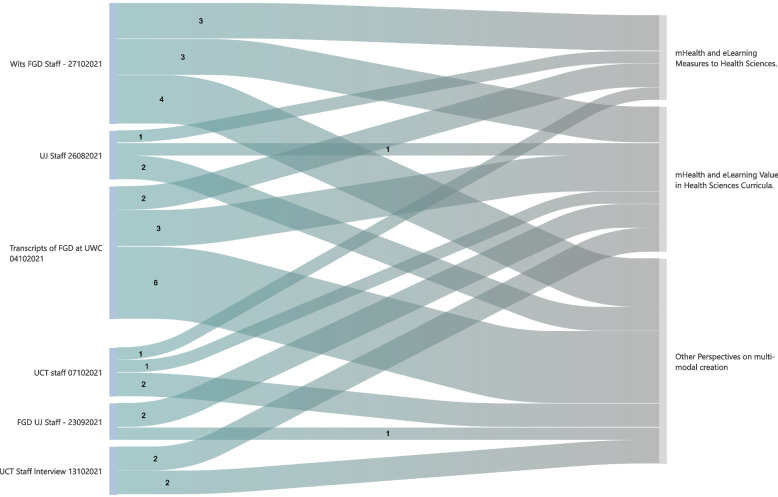
Perspectives on mHealth and e-Learning Measures and Value to Health Services

**Fig. 4:**
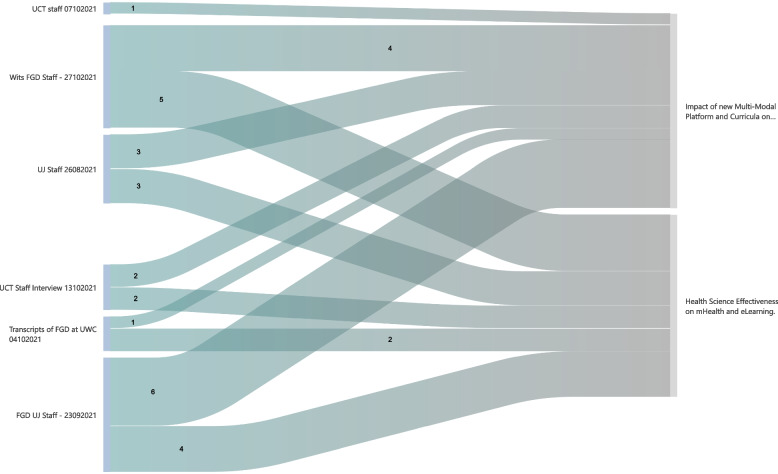
Impact of New Multi-modal Platforms and Health Sciences Effectiveness on mHealth and e-Learning

**Fig. 5:**
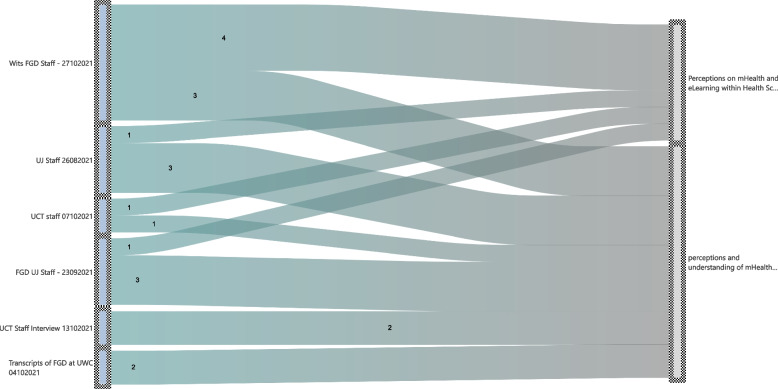
Perceptions of e-Learning and mHealth (as Multi-modal)

The findings consist of quantitative and qualitative data. The quantitative data provides information regarding the number of codes, by institution, per guide question. They are presented in Tables 1 - 8 in the Appendix section. Sankey diagrams were used to indicate the density of responses per guide question, by institution. They are presented in Figures 1 - 5 in the Appendix section. Since this study is not focused on a comparative analysis between institutions, the thematic findings per guide question presented below will not be differentiated by institution.

### Perceptions and understanding of mHealth and e-Learning within health sciences education

Guide Question 1: What are your perceptions and understanding of mHealth and eLearning within Health Sciences Education?

**Table:** 

Researcher Categories	Codes
Merging databases or datasets into mobile technology; Patient-focussed	**mHealth:** computational diagnostics; health-related information on digital applications: Examples given: Discovery applications recording health and testing fitness & testing hearing; electronic devices or applications to connect and share information and communicate; mobile devices and digital connected into one; differentiate mHealth from eHealth; mHealth is patient-focussed for interactive consultations and storage for identification of disease and treatments; eHealth is a broader practice such as Telemedicine or Telehealth
Merging electronic devices for Teaching and Learning; Student-focussed	**mHealth and eLearning:** applications for stimulating student learning – example given “virtual for rock”; distributed clinical practice learning and integration of theory
Limitation of e-Learning and necessity of integration	**eLearning:** restricted to 3D Visualisation; future need for haptic sensation; needs to integrate both into the curriculum

Participants have varied understandings and numerous comments related more to benefits and usefulness than actual definitions or descriptions of the two modalities, as evident in the codes above and Sankey diagram, Fig. 1 in Appendix. Fewer participants commented on mHealth and most commented on both modalities and the need to merge them. The need to merge the two modalities was articulated mainly in relation to enhancing teaching and learning as well as facilitating clinical practice in remoter areas. Those with more experience of mHealth referred to the merging of databases or datasets with digital technology and provided numerous examples. One participant differentiated between mHealth and Telemedicine or Telehealth, recognising the latter as encompassing a wider practice than an individual health practitioner consulting their mobile device for a narrower purpose, such as assisting with diagnosis, or testing hearing. One participant articulated the limitation of e-Learning as being confined to 3D visualisation and future developments needing to include haptic sensation, signally the differential requirements of professions. It would seem that mHealth is currently seen as patient-focussed and e-Learning as student-focussed. The selected quotations illustrate to some extent the variability amongst participants:

“mHealth primarily is mobile health technologies. It’s mainly used in terms of mobile computing or using mobile phones in terms of patient interactions, or different types of storage or is the identification of diseases or treatments”…*.*

*“e-Learning is more of an application than a teaching process … as educators, we are more in the realm of e-Learning, where students can be taught methods and application”...*

*“mHealth has got a bit of a software connotation for me. And the mobile and the digital sphere are all connected into one. e-Learning is more in the educational sphere, but it’s very much the same thing where it’s got a digital component and is associated with e-Learning”...*

“*mHealth is any based thing that you could do via your mobile phone and any other application that falls within the health sector. It is a vast scope that can be implemented within the Health Sciences education sphere*”…

*“Mobile applications and our e-Learning management system to augment and facilitate improved learning of their theoretical and clinical skills and knowledge”...*

### Health sciences engagement with mHealth and e-Learning

The second guide question: Do you think Health Sciences have been engaging effectively with mHealth and eLearning?

**Table:** 

Researcher Categories	Codes
Low effectivity due to limited or non-use	Does not exist for some disciplines; very limited use due to constraints; not relevant to South African Health; not compelling enough or applicable for all aspects of health care
High effectivity	Limited use but very effective: incorporation of videos for insightful learning
Enables distributed access	When used, provides demographic reach; can be taken into rural clinical services and teaching; insufficient usage for many reasons
Enhance learning	Integration of theory into practice-based learning
Multi-modal learning	mHealth and e-Learning need to be merged and integrated into the curriculum
Receptiveness	Readiness for Multi-modal learning
Scepticism	Question how useful it is for student learning

Table 2 and Figure 2 in Appendix provide quantitative data. Most agreed that health sciences have been engaging effectively with e-Learning, and have experienced the benefits it has for student learning. mHealth was rarely used, and some reported no usage.

It is evident from the codes that the meaning of engaging effectively with either mHealth or e-Learning, or both, has not been clarified explicitly. However, extent of use is a proxy for indicating support or endorsing either or both modalities. It is evident that the potential benefits of both are understood in terms of increasing access, enhancing learning and the value of integration of both; but these benefits have not been realised due to absence of integration which is a function of multiple constraints.

“*No, it’s not implemented as it should be, nor does it have much impact on the Health Sciences student education. The application should be one of the most reformed domains with four IR, starting with this student. And unfortunately, I think this, particularly within our context at our university, it’s not implemented” …*

*“I have only taught health sciences; the applications have not been practical enough, not all students can pay for the services. However, if the proposed applications are developed, it will be suitable to get biokinetics applications to the rural areas; this application will be perfect. However, data will be a challenge” …*

However, some participants agreed that the two platforms have been effective and are beneficiaries of the advanced 4IR platforms:

*“To some extent, the videos I have used has been very effective. Examples are Pixar applications with excellent, insightful videos that enable students to have a virtual parameter on student learning. However, since the students have limited time in practical classes, it is essential to access these applications at the beginning level to understand the importance of practice”…*

### Effectiveness of mHealth and e-Learning in health services

Guide question 3: How effective has mHealth and eLearning been in contributing to Health Services?

**Table:** 

Researcher Categories	Codes
Highly valued	mHealth: frequent use by professionals and students using their mobile devices
Pandemic-induced appreciation	Pandemic-induced necessity of blended learning increased frequency of use
Limited effectivity due to multiplicity of constraints	Limited effectivity due to lack of institutional response; limited student engagement; limited availability of technology and infrastructure in rural areas; limited value accorded by tutors and students due to constraints; ‘fear of the unknown’

As in the case of exploring effectiveness in health sciences *education*, what effectiveness means in relation to the health *services* is not discussed. Here too, extent of use is a proxy for indicating support or endorsing either or both modalities. Figure 3 in Appendix indicates that the majority of participants affirm neither platforms have been effectively engaged.

The great value attached to mHealth was conveyed by some participants:

*“Mobile health applications are frequently used by health professionals in the services … and students regularly use their mobile devices”….*

Others commented that the two modalities used in combination was induced by the COVID-19 pandemic, and they found this experience beneficial and stressful at times.

Several participants considered the either or both to have been of limited effectiveness for a variety of reasons, as indicated in the codes above:

*“Most students have not had a smooth ride with eLearning as sometimes they report to rural clinics or health centres where there is no network to communicate or utilise e-Learning platforms”…*

*….”not compelling enough; there is no student engagement”….*

### Challenges or factors confining health practitioners from utilising mHealth and e-Learning platforms

Guide Question 4: What challenges or constraints have been constraining health practitioners from utilising mHealth and eLearning platforms or why the unsuccessful rate of e-Learning within Health Sciences Education?

**Table:** 

Researcher Categories	Codes
Technological	Lack of connectivity and infrastructure; poor WiFi; insufficient data; lack of equipment and devices; applications developed internationally – not for Africa
Digital literacy limitations	Lack of technical skills amongst staff; technological generation gap between staff and students; some students lack digital skills; lack of Research Data Management skills
Unfocussed student learning behaviour	Limited student engagement due to online distractions
Socio-economic	Under-resourced settings; digital divide; lack of funding
Policy absence	Absence of adoption and regulation

Whether health practitioners in the health services or educators in health science faculties, similar constraints are reported. They range from limited or absent infrastructure and hardware, resulting in a digital divide that extends to skills of staff and students, which is largely a function of socio-economic inequality and absence of policy.

Furthermore, challenges vary according to discipline and types of activities engaged:

*“… in my profession, we do many simulations in practical activities. Now simulation is not something you can do on a smartphone. Still, we’re starting to also see the emergence of virtual reality in emergency care in general by allowing students to interact. We currently have a PC-based system, like a simulation system, presenting students with a case. The system enables the students to a range of treatment options and can practice their decision-making in a dual variety of things”…*

In addition to a paucity in technology, digital knowledge and skills, there is the complexity of contextual relevance:

*“There should be enough e-Learning and mHealth applications, but they are not available. Practitioners, as well as educators, are still lacking in this aspect. Secondly, I think there are all of these fantastic things and, like Frostine, applications in developed economies. Still, it’s not for us in Africa, so most are developed internationally. Also, lack of funding and ignorant on digital applications is a limitation confining the health sciences. Lastly, another rule might be changing the world, challenging successful implementation”…*

### mHealth and e-Learning impact if integrated into health sciences curricula and future practices

Guide Question 5: Do you think mHealth and eLearning will be valuable if integrated into Health Sciences curricula and future practices? Reasons.

**Table:** 

Researcher Categories	Codes
Hybrid approach	Only if it is a mixed approach that includes contact with patients
Strong support	Practitioners, staff and students will all benefit; access to care, teaching and learning in rural areas

As evident in Figure 4 in Appendix, most participants agreed that mHealth and e-Learning would be valuable if integrated into health sciences curricula and future practices, as all stakeholders would benefit, and in particular, distributed patient care and learning would be enhanced. Several emphasised the necessity of a hybrid approach, given that in many instances, patient care requires face-to-face contact.

The following two quotes indicate support but uncertainty about outcomes and impact:

*“Until we implement all these technologies at our disposal and implement them within our teaching, we obviously won’t know the impact it has on the students. Both staff and students should be comfortable using these applications and get rid of the fear of the unknown. If mHealth and e-Learning are integrated into the curricula, teaching and learning will be more accessible in health sciences”…*

*“Yes, it may add value, but how practical, I cannot say at this moment as some things could be good, not excellent or counter-productive, but until it is implemented, the outcome cannot be predicted”…..*

Another participant went further, and observed: *“yes, a unified platform will be a future and potentially where we will have to incorporate machine learning systems”*…

### Transferable skills to be embedded with the future curriculum using e-Learning and mHealth

Guide Question 6: What transferable skills ban be embedded within the future curriculum using e-Learning and mHealth?

**Table:** 

Researcher Categories	Codes
Social	Communication
Strengthening Research	Research Data Management; Data Analytics
Technical	Technical/digital skills
Advanced Learning	Learning Design and 21st Century Learning Skills
Attitudinal or Disposition	Open-mindedness and willingness to embrace technology

A range of transferable skills from social to attitudinal were identified as indicated in the codes above. Most frequently cited were communication and digital skills (for practitioners, staff and students), followed by those strengthening research and advance learning. One participant emphasised appropriate attitude given experience of colleagues not taking e-Learning seriously, viewing it more as support in current circumstances (pandemic context) which may be phased out in future.

### Devices used when accessing technology platforms or learning management systems

Guide Question 7: Which device do you most often use when accessing technological platforms or learning management systems; and which device do you prefer using in your spare time when not working/studying; and which device for study or work?

**Table:** 

Researcher Categories	Codes
Mobile Digital	Laptop (most frequently cited); mobile phone (used to a lesser extent); Laptop combined with mobile phone; i-Pads; Tablets
Learning Management Systems	Blackboard; Sakai and VULA; Canvas; Moodle

It was evident from the discussions and Table 6 in Appendix that the majority of participants used laptops most frequently, whether for work or study as well as for accessing their LMS. Furthermore, LMS varied between institutions with some participants being familiar with more than one LMS.

Most participants reported that all first-year student receive tablets for their academic work and e-books, which has made education much easier for the students. The main reason for this policy is that less privileged students cannot afford a laptop. Hence the tablets are seen as a mode of communication that enhances student learning.

### Effective mediums and platform options that allow the bridging or optimal balance of mHealth and e-Learning to advance teaching and engagement for health sciences education

Guide Question 8: What mediums and platforms would be most effective in bridging or providing optimal balance between mHealth and eLearning to advance teaching and engagement for Health Sciences Education?

**Table:** 

Researcher Categories	Codes
Integration of LMS with relevant applications	Integrating Teams Classroom and LMS; integrating applications and LMS; integrating applications to access videos
Hybrid	Contact learning combined with Multi-modal
Fit-for-purpose	Variable platforms each with own strengths and weaknesses to be evaluated for integration decision-making; infrastructure needs to support learning pedagogies
Early exposure to Multi-modal platforms	Multi-modal needs to be started early in students’ learning
Policy	Require explicit goal of optimal balance of mHealth and e-Learning pedagogies

Figure 2 in Appendix presents the codes that emerged from the FGD and interview findings.

Variability was evident among the participants. Some focussed on integration of LMS with applications, others emphasised the necessity of a hybrid approach that incorporates face-to-face learning and patient care; yet others recommended a fit-for-purpose approach:

“*There are different innovative applications integrated into teaching and learning within the institutions. Educational learning management systems, Dictionary App, developing a textbook on a mobile app amongst many. Students need this on their learning and practical activities, which will be great if added to the platform that is to be developed. Some current applications are developed to check for eye defects”…*

*“It is essential to build the infrastructure that supports these learning pedagogies. There are specific platforms such as VULA, Blackboard, Canvas, Viewmodels, and several others. Each of these platforms or systems comes in with its positives and its limitations. So the question is about how to strike a balance”…*

Another participant commented that.

“*platforms such as telemedicine, the 4IR, and the AI are all already with us and not just the future”…*

and that students should have early exposure as well as work with those platforms relevant to the field in which they will practice. This will also require a policy framework. Fit-for-purpose and digital literacy were emphasised by most participants.

### Perspectives on optimising both learning and healthcare in South Africa through multi-modal platforms

Guide Question 9: Do you believe a multi-modal platform and curricula that have been evaluated and developed will optimise both learning (for students and academic health staff) and healthcare (patients) in South Africa?

**Table:** 

Researcher Categories	Codes
Contingent support; Strong clinical focus	If learning can be translated into clinical outcomes
Hybrid	If the platform communicates across patients, students and e-Learning-type activities and provides for face-to-face; Multi-modal is necessary for categories of healthcare that require face-to-face
Performativity and Quality Assurance	Existing platforms require effective functioning; phones to access/interface with LMS is vital
Efficiency	Existing platforms that can be integrated into Teaching and Learning; one app to serve integrating function; will save time for future clinicians
Relevance, appropriateness, affordability	New applications need to be low tech, low grade, mobile, user-friendly
Policy for implementation	Needs to be guiding principles and framework on what to do; people need to be brought together to understand what needs to be done
Digital Policy Guidance; Adequate high-level resourcing	Role of government and telecommunications organisations necessary to advance integration into both Teaching/Learning and Service
Conditional	Concept of self-triage requires change in e-Learning and acceptance by health practitioners

The majority of participants were supportive of a multimodal platform (see Table 8 in Appendix), as well as conveying an understanding or appreciation of what will be required evident in the codes above. The benefit of efficiency was foregrounded and applied to various dimensions of the patient-care-teaching-learning spaces. Additionally, several observed that performativity, quality assurance, relevance, appropriateness and affordability were necessary considerations to achieve those efficiencies. Two participants experienced in integrated LMS and applications multi-modalities further endorsed the view of others that a policy for implementation of integrated multimodalities is a necessity, and requires adequate resourcing at a high or macro level that includes government and telecommunications companies. In summary, they indicate that all actors, stakeholders, hardware, software and infrastructure need to be integrated under one POLICY platform.

One participant’s comment highlighted the scale and depth of change required, attitudinally, in their referral to *“the concept of self-triage (patient autonomy and participation via mHealth) requires change in eLearning + acceptance by health practitioners”* which echoes an earlier comment regarding the types of transferrable skills required: *“open-mindedness and willingness to embrace technology”.*

Further selected quotations that convey some of the codes captured above:

*“Yes, I certainly do believe multi-modal will directly link to all the various issues in the health sector. For example, health practitioners can design and develop video-based learning and live interaction sessions or content that everyone can do in their own time. And they can develop a multi-modal where you get feedback from systems either through AR or VR that allows health practitioners to reduce the number of contact and practical sessions will be an added advantage. However, I don’t think this can be achieved soon. It will take a while before it can be accomplished” …*

Another participant agreed that,

*“It would be ideal if multi-modal platforms were integrated to make a smoother learning environment. But, still, at the same time, I think it’s essential that we don’t put too much into applications and platforms for the sake of having all this technology” …*

However, the participant indicated that students might already feel overwhelmed and bombarded with so much that if more platforms are added for the sake of adding in, it might be too much on them and lose its effectiveness. Hence, educators and the people that designed these platforms must decide what needs to go specifically and make sure that anything that does go into such a platform or the curriculum is beneficial to the students. In addition, students must be already exposed to these new applications before they graduate to make life easy for them. However, a participant had a different opinion by stating.

*“Despite embracing all the technology in the world, if students are not taught soft skills and how to care for patients, they will not have in-depth experience on improved patient care experience. Hence, it would be great to integrate both models and see their value to teaching and learning in the health sciences” …*

In conclusion, Figure 5 in Appendix, conveys that the FGDs and interview commenced with majority support for mHealth and e-Learning despite various understandings of each, and by the end of the FGDs, the majority extended their support specifically for integrating the modalities as they considered them beneficial to patient-care, student learning and staff teaching. A minority were sceptical but not opposed given the level of resourcing, planning and coordination required; and some emphasised the necessity of multi-modal including face-to-face contact with patients given the sensitivity and complexity of some of the consultations.

## Discussion

Given the limited uptake of e-Learning within the health sciences area in South Africa, this study aimed to fill this gap by researching staffs’ perceptions and understanding of e-Learning and mHealth, the value accorded the two applications when combined into a multi-modal platform, as well as constraints and possibilities for the future. Findings relating to perceptions of all the staff members on e-Learning and mHealth indicate all participants are either aware of both mHealth and e-Learning or familiar with both. However, most believe that e-Learning has been embraced more than mHealth. While supportive, some expressed reservations about how effective e-Learning was for students’ learning given the challenges discussed below.

Most participants agree that mHealth applications are fully embraced within the health sector which requires reciprocal responses from the educators and trainers. The analysis corresponds with the literature that “mHealth Education” or “mHealthEd” are a new set of mobile devices and applications that are used as support systems for optimising upskilling among individuals to enhance quality patient care, and if embraced, can assist health practitioners in testing, supporting and supervising health care workers, as well as the provision of health care information to individuals. Hence academic journals and a growing literature on mobile applications role in managing chronic conditions and preventive medicine, is paramount [25, 26].

On the theme of challenges and factors confining health practitioners from utilising mHealth and e-Learning platforms, a variety of perspectives emerged: resource inequalities, the digital divide, appropriateness to the discipline, as well as varied skill levels between staff and students which could all impact student motivation. Their comments concur with those of Regmi and Jones’ systematic review [27], and that of Ortega, Villalta, Rodriguez, Arpi et al. [28]. More specific to under resourced settings were lack of technical skills, connectivity issues, inadequate educational training, insufficient data for students, lack of Wi-Fi stability or infrastructure, lack of skills or education, the generational gap, all of which underscore the concept of the digital divide. These findings concur with those of Garad, Al-Ansi and Qamari [5].

One of the objectives for mHealth and e-Learning is to beneficially impact learning via integration into the health sciences curricula and future practices. All participants agree that both applications are the future and will be a valuable contribution in optimising the teaching and learning curricula in health sciences. The scepticism of one participant is related to whether or not adequate resourcing will be available in a fit-for-purpose approach to integrating the modalities. Most believe that transferable skills such as research data management skills, communication skills, technical skills and data analytics skills are essential tools needed to understand and operate the two applications. The latter finding is also widely reported in the literature [5, 11].

Participants reported varying Learning Management Systems (LMS) used amongst the universities, for example, Blackboard, Moodle, Sakai, Vula and Canvas. Common to all was the use of laptops and mobile devices. This finding of LMS being augmented with mobile digital devices concurs with the literature [11, 5]. It was further suggested that effective mediums and platform options that allow the bridging or optimal balance of mHealth and e-Learning should be developed to advance teaching and engagement for health sciences education. Some participants provided examples of how they were trying to achieve this, for example, the addition of videos for first years to the LMS, integration of the Dictionary App, diagnostic applications, and textbooks on mobile devices. Others also referred to Telemedicine. If there was to be effective and efficient service delivery to all health practitioners and patients within the country, collaboration and policy was necessary to develop a multi-modal platform that took account of performativity, quality assurance, relevance, appropriateness and affordability.

These findings concur with the literature and highlight the need for urgent technological innovation within health sciences to bridge the gap left by COVID-19 on utilising e-Learning and mHealth to optimise the health sciences curricula. e-Learning has been beneficial during the pandemic, as confirmed by Ortega, Villalta, Rodriguez, Arpi et al. [28], but at the same time, there has been a challenge in terms of lab work, practicals or clinical-based competencies wherelearning could not be addressed effectively. Similar findings relating to STEM disciplines were reported by Al-Ansi [29]. Evidence has shown progress in developed economies on mHealth and e-Learning that would be useful if integrated within various curricula so that health practitioners (both staff and students) do not miss out on their clinical competency training.

It is, thereby, suggested that a multi-modal platform would enable an offering in an offline mode in the rural areas, where there is not adequate Wi-Fi connection. Ideally, the platform would work offline and access all various patient records on the system. The unified approach might prove to be helpful. However, every participant agreed that the adoption would take time due to the various challenges that persist within different universities and the socio-economic landscape.

### Limitations of this study (as a component of the larger research project)

This study has a number of limitations. During the pandemic, it was logistically challenging to have all FGDs and the interview conducted in person. Having it in person would have allowed the researchers to elucidate additional cues from the answers given by participants. In addition, the COVID-19 lockdown prevented more FGDs and interviews taking place, due to some of the restrictions imposed for staff being involved in research, at other universities. However, this did not impede on the required sample number of staff in this study, in order to derive meaningful insight and understanding of the topic at hand.

## Conclusions

The study demonstrated that most of the participants agreed that mHealth and e-Learning could be integrated for health sciences education and health care professionals. This can also benefit students if exposed to mHealth (or Extended Realities - XR) in universities and adopted in the clinical setting. With the new generation of health professionals, some skills such as diagnosing and treating an array of diseases has become complex and challenging. Most participants believed that these broad spaces could integrate mHealth and e-Learning. However, among the various challenges addressed are the social-economic difficulties that produce a digital divide, such as insufficient data for students or lack of Wi-Fi stability or infrastructure, load (electricity) shedding in South Africa, poverty as well as digital illiteracy. Therefore, it is essential to upskill both students and staff on digital literacy. Suggestions were further raised on the transferrable skills for healthcare professionals transitioning into an industry where data analytics and data management are being managed as well as gaining a comprehensive understanding of analysing data. Lastly, staff members agree that a new multi-modal platform (in the form of a learning management system with relevant and appropriate applications) tailored towards health sciences will benefit all stakeholders and be valuable to higher education and health sectors. Change is inevitable and change should be adopted within health sciences. Digitalisation and digital citizenship are gradually being integrated into teaching and learning. Now is the time to streamline and adapt health sciences curricula to promote health sciences education in the current 4IR.

## Data Availability

The datasets generated during and/or analysed during the current study are not publicly available due to the confidential nature of participants’ perceptions and experiences. However, the data (through anonymity) is available from the corresponding author on reasonable request.

## Appendix

**Table 1:** Perceptions and understanding of mHealth and e-Learning within Health Sciences Education

	○ Perceptions and understanding of mHealth and e-Learning within Health Sciences Education Code Themes = 18	○ Perceptions on mHealth and e-Learning within Health Sciences Education Code themes = 8	Totals
FGD UJ Staff - 23092021	3	1	4
FGD at UWC 04102021	2	0	2
UCT staff 07102021	1	1	2
UJ Staff FGD 26082021	3	1	4
Wits FGD Staff - 27102021	3	4	7
UCT Staff Interview 13102021	2	0	2
Totals	14	7	21

Table 1 below and the Sankey diagram below (Fig. 1) reflect the theme’s emerging codes. In addition, there were four perceptions codes from FGD in UJ, two from UWC, two from UCT, another four from UJ FGD, seven from WITS and two from the UCT staff interviews. All these codes were analysed and integrated into the discussions below.

From the focus group discussions, there were 16 quotations on mHealth and e-Learning perceptions within health sciences and eight perceptions on its relevance within health sciences education.

Table 2 and Fig. 2 reflects Health Sciences effectiveness in mHealth and e-Learning; in which four codes themes arise from the focus group with UJ staff, two from UWC, one from UCT Staff Interview, three from UJ staff FGD, five code themes came from Wits staff members and two from UCT staff interviews. Figure 2 below shows the number of codes received by the staff focus group discussions on each quotation (Fig. 3) (Table 3).

**Table 2:** Health Sciences Engagement with mHealth and e-Learning and the value of a Multi-Modal Platform

	○ Health Science Effectiveness on mHealth and e-Learning Codes = 25	○ Impact of new Multi-Modal Platform and Curricula on Health Sciences Codes = 18	Totals
UJ FGD Staff – 23092021	4	6	10
UWC FGD Staff - 04102021	2	1	3
UCT staff interview 07102021	1	1	2
FGD UJ Staff 26,082,021	3	3	6
Wits FGD Staff - 27102021	5	4	9
UCT Staff Interview 13102021	2	2	4
Totals	16	17	34

**Table 3:** Challenges or factors that have been confining health practitioners from utilising mHealth and e-Learning platforms

	○ Challenges or Factors Confining Health Practitioners from utilising mHealth and e-Learning Platforms Code themes = 55
FGD UJ Staff - 23092021	Online distractions, No student engagement, Loadshedding, Wi-Fi network, Generational Gap.
Transcripts of FGD at UWC 04102021	Internet access, No student engagement, Insufficient data.
UCT staff 07102021	Lack of technical skills, digital divide, connectivity issues, lesser resourced settings, inequality, inadequate educational training, insufficient data for students, lack of Wi-Fi stability of infrastructure, lack of skills or education.
UJ Staff 26082021	Insufficient preparation from both tutors and students, poor backgrounds, lack of accessibility to data, Adoption and regulation.
Wits FGD Staff - 27102021	Online distractions, no student engagement, loadshedding, Wi-Fi network, Generational Gap.
UCT Staff Interview 13102021	No digital training, connectivity issues and systemic, digital divide, load-shedding.

**Table 4:** mHealth and e-Learning impact (if integrated into health sciences curricula and future practices)

	○ mHealth and e-Learning Measures to Health Sciences. Code themes = 7	○ mHealth and e-Learning Value in Health Sciences Curricula. Code Themes = 22	○ Other Perspectives on multi-modal creation Code Themes = 17	Totals
FGD UJ Staff – 23,092,021	0	2	1	3
UWC FGD Staff - 04102021	2	3	6	11
UCT Interview staff 07102021	1	1	2	4
UJ Staff 26,082,021	1	2	2	5
Wits FGD Staff - 27,102,021	3	3	4	10
UCT Staff Interview 13,102,021	0	2	2	4
Totals	7	13	17	37

In Table 4, 12 codes emerged: two from UJ staff, three from UWC, one from UCT interview, two from UJ FGD, three from WITS, and two from UCT.

From Fig. 4, several codes emerged from the staff FGDs and interviews. On the concept of the impact of the new model on themes. Four themes emerged from Wits FGD, three codes from UJ Staff FGD, two from UCT FGD, one from UWC interview, one from UCT Staff and six themes from UJ FGD. However, from the concept of health science effectiveness on mHealth and eLearning, five codes emerged from Wits FGD, three code themes from UJ Staff FGD, two codes from UCT FGD, four codes on UJ FGD and one code from the UWC interview (Table 5).

**Table 5:** Sequence of transferable skills embedded with the future curriculum using e-Learning and mHealth

	Transferable skills
FGD UJ Staff - 23092021	Technological skills, Communication skills.
Transcripts of FGD at UWC 04102021	Education and Training skills, communication skills.
UCT staff 07102021	Research Data Management skills, communication skills, technical skills, data analytics skills.
UJ Staff 26082021	Communication skills, technological skills, learning design and twenty-first-century learning skills scores, and twenty-first-century learning skills scores.
Wits FGD Staff - 27102021	Statistical analysis, data analysis, E-portfolio of learning, communication, constructive writer and critical thinker.
UCT Staff Interview 13102021	Data Management Skills.

**Table 6:** Devices used in accessing technological platforms or learning management systems

	○ Device Used in Accessing Technology Platforms for Study or Work Code themes = 37	Types of Devices and Platforms	Totals
FGD UJ Staff - 23,092,021	8	Laptops, Mobile, Sakai, Moodle	8
Transcripts of FGD at UWC 04102021	3	Vula, Canvas, Mobile, Laptops	3
UCT staff 07102021	2	Vula, Canvas, Mobile, Laptops	2
UJ Staff 26082021	2	Blackboard, Mobile phones, Laptops	2
Wits FGD Staff - 27102021	7	Sakai, Moodle, Mobile Phones, Laptops	7
UCT Staff Interview 13,102,021	2	Vula, Canvas, Mobile, Laptops	2
Totals	24		24

Table 6 presents different devices and the number of codes that emerged from each theme in accessing technology platforms or learning management systems in the four universities. For example, UJ staff members are familiar with Blackboards, mobile phones and laptops. However, some members are familiar with Vula and Sakai platforms. UCT and UWC staff members are familiar with Vula and Canvas platforms, while Wits uses Sakai and Moodle alongside mobile phones and laptops.

**Table 7:** Effective Mediums and Platform Options to Advance mHealth and e-Learning

	○ Effective Mediums and Platform Options to Advance mHealth and e-Learning Code themes =15	○ Effective Platform Options for Optimal Balance on mHealth and e-Learning Code Themes = 18	Totals
FGD UJ Staff - 23092021	0	3	3
Transcripts of FGD at UWC 04102021	3	4	7
UCT staff 07102021	1	2	3
UJ Staff 26082021	1	1	2
Wits FGD Staff - 27102021	2	1	3
UCT Staff Interview 13102021	1	2	3
Totals	8	13	21

Table 7 reflects the number of codes that emerge from each focus group discussion. Three codes emerged from effective mediums and platform options to advance mHealth and e-Learning. Seven came from FGD at UWC. Three appeared from UCT, two came from the second FGD in UJ, three emerged from WITS FGD and UCT staff interviews.

**Table 8:** Perceptions of e-Learning and mHealth (as Multi-modal)

	○ Impact of new Multi-modal Platform and Curricula on Health Sciences Code themes - 17	○ Other Perspectives on Multi-modal creation Code themes - 17	Totals
FGD UJ Staff – 23,092,021	6	1	7
Transcripts of FGD at UWC 04102021	1	6	7
UCT staff 07102021	1	2	3
UJ Staff 26,082,021	3	2	5
Wits FGD Staff – 27,102,021	4	4	8
UCT Staff Interview 13,102,021	2	2	4
Totals	17	17	34

Table 8 presents the findings from the perceptions of eLearning and mHealth. It translates the codes emerging from the Sankey diagram. The number of codes emerging highlights the understanding of the staff members in the universities sampled on eLearning and mHealth, specifically the multimodal system.

From the above figure (Fig. 5), seven themes came from the Wits FGD on the perceptions of mHealth and eLearning, while the UJ staff FGD has 4 codes, and the UCT FGD had 3 codes. The second UJ FGD had 4 codes, the UCT staff interview had 2 codes and the UWC FGD also had 2 codes emerging on the understanding of mHealth and eLearning. The codes are translated in Table 8 above.

## Abbreviations

4IR: Fourth Industrial Revolution
COVID-19: Coronavirus-19
FGD: Focus Group Discussion
FGDs: Focus Group Discussions
ICTs: Information Communication Technologies
LMIC: Low-middle income countries
mHealth: Mobile Health
mHealthEd: Mobile Health Education
UCT: University of Cape Town
UJ: University of Johannesburg
UWC: University of Western Cape
Wits: University of the Witwatersrand
WHO: World Health Organisation
